# Occult tethered cord syndrome: insights into clinical and MRI features, prognostic factors, and treatment outcomes in 30 dogs with confirmed or presumptive diagnosis

**DOI:** 10.3389/fvets.2025.1588538

**Published:** 2025-07-11

**Authors:** Javier Espinosa Romero, Steven De Decker, Koen Santifort, Rodrigo Gutierrez-Quintana, Maria Ortega, Ane Uriarte, Abtin Mojarradi, Quinten van Koulil, Despoina Douralidou, Irene Espadas, Miguel Benito Benito, Carlo Anselmi, Charlotte Dye, Patricia Alvarez, Juan José Minguez, Abbe Crawford, Christoforos Posporis

**Affiliations:** ^1^Neurology and Neurosurgery Department, Independent Vetcare (IVC) Evidensia, Pride Veterinary Referrals, Derby, United Kingdom; ^2^Department of Clinical Science and Services, Royal Veterinary College, Hatfield, United Kingdom; ^3^Neurology and Neurosurgery Department, Independent Vetcare (IVC) Evidensia, Dierenziekenhuis Arnhem, Arnhem, Netherlands; ^4^Neurology and Neurosurgery Department, Independent Vetcare (IVC) Evidensia, Dierenziekenhuis Hart van Brabant, Waalwijk, Netherlands; ^5^Small Animal Hospital, School of Veterinary Medicine, University of Glasgow, Glasgow, United Kingdom; ^6^Neurology and Neurosurgery Service, Anicura Indautxu Hospital Veterinario, Bilbao, Spain; ^7^Neurology and Neurosurgery Department, Southfields Veterinary Specialists Part of Linnaeus Veterinary Limited, Basildon, United Kingdom; ^8^Neurology and Neurosurgery Service, AWAKE Animal Hospital, Helsingborg, Sweden; ^9^Neurology and Neurosurgery Department, The Ralph Veterinary Referral Centre, Marlow, United Kingdom; ^10^Neurology and Neurosurgery Service, Unavets Veterios Referral Hospital, Madrid, Spain; ^11^Anderson Moores Veterinary Specialists, Part of Linnaeus Veterinary Limited, Winchester, United Kingdom; ^12^Imaging Department, Independent Vetcare (IVC) Evidensia, Blaise Veterinary Referral Hospital, Birmingham, United Kingdom; ^13^Internal Medicine Department, Independent Vetcare (IVC) Evidensia, Pride Veterinary Referrals, Derby, United Kingdom

**Keywords:** conus medullaris, dural sac, filum terminale, pain, incontinence, behavior, lumbosacral, dynamic MRI

## Abstract

Occult tethered cord syndrome (OTCS) is poorly documented in dogs. This retrospective multicenter study evaluated the clinical presentation, MRI findings, treatment outcomes, and prognostic factors in 30 dogs diagnosed with OTCS managed surgically (*n* = 11) or medically (*n* = 19). Novel clinical severity scoring and neurological grading systems were developed to assess prognostic utility. The median age at clinical onset was 11 months (range 2–65), with a median duration of clinical signs of 13 months (range 1–60). Pain/dysesthesia in the lumbosacral region/tail/pelvic limbs was the most common presenting complaint (97%), followed by pelvic limb gait abnormalities (70%), behavioral changes (67%), impaired physical activity (63%), and urinary/fecal incontinence (17%). Neurological deficits were present in 90% of dogs. MRI findings showed variability in conus medullaris and dural sac termination, with no physiological translocation detected in available dynamic studies. Electrodiagnostic abnormalities were identified in four of nine tested dogs (44%). Clinical severity scores strongly predicted response to medical treatment, with responders having significantly lower scores than non-responders (3.25 ± 2.09 vs. 7.78 ± 3.15, *p* < 0.001). Higher neurological grades (*p* = 0.006), presence of behavioral abnormalities (*p* = 0.045), and worsening clinical evolution prior to referral (*p* = 0.009) were also associated with poor medical therapy outcomes. Surgical intervention was significantly associated with full recovery (*p* = 0.015) and discontinuation of medical treatment (*p* = 0.023) at last follow-up (median: 9 months, range: 2–108). Three surgically treated dogs experienced partial relapse within 6 months, with two undergoing reintervention and improving postoperatively. This study highlights the clinical and MRI characteristics of canine OTCS, introduces novel prognostic factors, and supports surgical detethering as a key intervention for optimizing outcomes. Larger prospective studies are needed to validate these findings, refine the proposed scoring systems, and establish evidence-based guidelines for managing canine OTCS.

## 1 Introduction

Tethered cord syndrome (TCS) is a rare congenital or acquired condition caused by abnormal caudal positioning and/or traction of the spinal cord and meninges, predominantly observed at the level of the conus medullaris (CM) (1, 2). Congenital TCS may result from a tight filum terminale (FT), characterized by reduced elasticity, shortened length, and/or abnormal composition, and may coexist with malformations arising during primary or secondary neurulation (3). In dogs, TCS has been reported in association with spina bifida, myelomeningocele, myeloschysis, and dermoid sinus type VI (4–8). However, it may also occur without overt structural deformities. The term occult tethered cord syndrome (OTCS) has been proposed in such cases, where clinical signs are suggestive of the condition, yet no structural abnormalities are identified on magnetic resonance imaging (MRI) (9–11).

The underlying pathophysiology of canine OTCS remains undetermined. In humans, a structurally abnormal, short, and/or inelastic FT exerts excessive caudo-dorsal traction on the CM and nerve roots (12), resulting in impaired neurovascular function, hypoxia, secondary metabolic disturbances, and progressive neurological deficits (13). A similar mechanism is suspected but has not been definitively proven in dogs (14). Moreover, the absence of detailed anatomical and histological descriptions of the normal FT in dogs limits our ability to identify structural deviations and impedes understanding of OTCS pathogenesis. In humans, among scarce morphometric studies, a recent cadaveric investigation has provided valuable insights (15). Analogous studies in dogs could help clarify the factors contributing to this condition.

Reported clinical signs include pelvic limb weakness and/or lameness with or without proprioceptive deficits, urinary incontinence, low carriage and reduced tone of the tail, reduced withdrawal reflexes in pelvic limbs, sensory abnormalities with pain-related behaviors and lumbosacral pain on palpation. These neurological abnormalities are typically indicative of a neuroanatomic localization of L4–S3 spinal cord segments or cauda equina (16, 17). However, the exact spectrum of neurological signs, their prevalence, and clinical evolution over time remain poorly characterized in veterinary medicine.

Dynamic lumbosacral MRI may aid OTCS diagnosis by evaluating physiological CM translocation, with a fixed position increasing suspicion in the absence of alternative diagnoses (14, 18–20). However, objective criteria for selecting surgical candidates remain undefined. In human medicine, clinical severity scales have predictive value (21) and a similar approach in dogs could enhance diagnostic accuracy and inform treatment decisions.

The presumptive diagnosis of OTCS can be confirmed by a positive response to surgical detethering following dissection of the FT, alongside supportive intra-operative findings, as previously reported in both human patients and dogs (9, 14, 16). Consequently, surgery serves a dual purpose, functioning as both a diagnostic tool and a therapeutic intervention. This raises a considerable ethical dilemma for practicing neurosurgeons, as the necessity of an invasive procedure can only be judged retrospectively.

In the authors’ experience, OTCS can be a debilitating, chronic, and likely underdiagnosed condition that significantly impacts quality of life. In humans, early surgical intervention is linked to improved outcomes, and similar successes have been reported in dogs (16, 17), highlighting the importance of establishing accurate diagnostic standards to ensure timely identification and appropriate management. This study aims to address the knowledge gap by characterizing the clinical presentation, MRI findings, treatment modalities, and outcomes in dogs with OTCS. It also seeks to identify prognostic factors to guide treatment decisions and predict outcomes, advancing the understanding of OTCS and supporting evidence-based approaches in veterinary medicine.

## 2 Materials and methods

A retrospective, descriptive, multicenter study was undertaken with cases recruited from the clinical databases of referral centers in the United Kingdom, Spain, the Netherlands, and Sweden. Ethical approval was granted (URN SR2024–0012; Social Science Research Ethical Review Board, Royal Veterinary College) prior to accessing any medical records or client communication for follow-up purposes.

### 2.1 Inclusion criteria

Client-owned dogs of any age, breed, sex, and neuter status with neurological signs consistent with L4-S3 spinal cord and/or associated spinal nerves/cauda equina neuroanatomical localization and an unremarkable static MRI (in neutral position) of the thoracolumbar, lumbosacral, and tail base regions were included in the study. All neurological examinations were performed by a board-certified or board-eligible neurologist. Dynamic MRI findings were evaluated but not required for inclusion. A subjectively dorsal CM and/or dural sac (DS) position or a caudal CM or DS termination (CMt, DSt) were not classified as abnormalities due to known variability across dogs of different body weights and breed (22, 23) and lack of established radiologic criteria in veterinary medicine.

A diagnosis of presumptive OTCS (P-OTCS) was assigned to dogs not undergoing surgical detethering. Surgically treated cases, involving dissection of the FT externum (FT*e*) and/or internum (FT*i*), were categorized as (a) responders, showing positive postoperative response (partial or full, sustained or temporary), or (b) non-responders, if no improvement occurred during follow-up. Responders to surgery were classified as confirmed OTCS (C-OTCS), while non-responders retained an open diagnosis unless FT pathology was confirmed on histopathology in which case they were reclassified as C-OTCS. Subjective intraoperative observations, such as a tight, short, or thickened FT and/or cranial displacement of the DS or CM following FT dissection, were recorded but not required for diagnostic confirmation. Given the absence of validated histopathological markers for OTCS in dogs, a positive surgical response was considered sufficient for clinical confirmation and classification as C-OTCS, regardless of histopathology availability or findings.

### 2.2 Exclusion criteria

Cases with abnormalities on physical or orthopedic examination explaining the clinical signs were excluded, as were those with significant MRI findings in the thoracolumbar, lumbosacral, or tail base regions, including the spinal cord and/or nerves. To enhance diagnostic confidence in the P-OTCS group, dogs who underwent dynamic MRI were removed if the study showed physiological CM translocation or dynamic lumbosacral vertebral canal and/or foraminal stenosis (24). This exclusion criterion was not applied to dogs with C-OTCS. Cases with lumbosacral vertebral stabilization, incomplete records, missing follow-up data, or follow-up shorter than 2 months were also excluded, along with those exhibiting clinical signs or electrodiagnostic/histopathological evidence of generalized or multifocal neuromuscular disease. Dogs with focal pelvic limb/lumbosacral/tail electrophysiological abnormalities were retained in the cohort.

### 2.3 Data collection

Clinical data included signalment (age, breed, sex, and neuter status), body weight, medical history, comorbidities, prior treatments, and findings from physical, orthopedic, and neurological examinations. MRI and additional diagnostic tests were reviewed. Cerebrospinal fluid (CSF) analysis was interpreted according to established reference values (25), as were the findings of electrodiagnostic investigations (26). Treatment details, intraoperative observations, follow-up assessments, and final outcomes were documented. A novel 16-point scoring system, adapted from recent human OTCS classifications (21), was developed to quantify clinical severity (Table 1). The scoring system was expanded to include behavioral modifications, reflecting anecdotal clinical experience indicating a high prevalence in affected dogs and known associations between lumbosacral disease and behavioral comorbidities in the veterinary literature (27). Based on assigned scores, cases were stratified into severity grades I–IV (Table 2).

**Table 1:** Clinical severity scoring system in dogs with OTCS adapted from Klinge et al. (21).

Clinical signs	Score^∅^
Neurological deficits*	
Posture	1
Gait	1
Postural reactions	1
Spinal segmental reflexes	1
Muscle mass/tone	1
Pain^	
Lumbosacral/tail base	1
Pelvic limb/s	1
Defecation/urination	1
Incontinence	
Fecal	1
Urinary	1
Behavior	
Aggression	1
Compulsion	1
Restlessness	1
Lethargy	1
Physical activity	
Intolerance	1
Reluctance^**^	1
Total	16

^∅^normal = 0, abnormal = 1.

^*^In pelvic limbs.

^Including suspected dysesthesia (paresthesia and/or allodynia).

^**^Able to exercise but unwilling/reluctant to do so.

**Table 2:** Clinical severity grading system in dogs with OTCS.

Neurological grade	Clinical severity score	T-OTCS	C-OTCS	P-OTCS
Grade I	1–4	12	2	10
Grade II	5–8	11	5	6
Grade III	9–12	5	2	3
Grade IV	13–16	2	2	0

T-OTCS, entire cohort of dogs with OTCS; C-OTCS, surgically managed confirmed OTCS group; P-OTCS, medically managed presumptive OTCS group.

All MRI studies were assessed by board-certified radiologists and neurologists. For the purpose of this study, images were re-evaluated jointly by the first and last authors together with the same board-certified radiologist. Independent assessments were not performed; therefore, intra- and interobserver agreement were not calculated. Morphologic findings were included in the analysis only when consensus among the three observers was reached. In cases where agreement could not be achieved, the findings were excluded and only the number of such instances was documented. Anatomical structures relevant to our study are shown in Supplementary Figure S1.

High-field (1.5 T) MRI scanners were predominantly used, with low-field (0.2 T) systems utilized less frequently. MRI planes and sequences varied by hospital protocol and case requirements. Sequences included T2-weighted, short tau inversion recovery (STIR), heavily T2-weighted, gradient echo, T2-weighted 3D fast imaging employing steady-state acquisition (FIESTA), and pre- and post-contrast T1-weighted or fat saturated T1-weighted sequences, acquired in sagittal, transverse, or dorsal planes as determined by the leading clinician. Post-contrast images were obtained using Gadoterate Meglumine (Dotarem R, Guerbet, UK; Clariscan, GE Healthcare, Australia).

**Figure 1:**
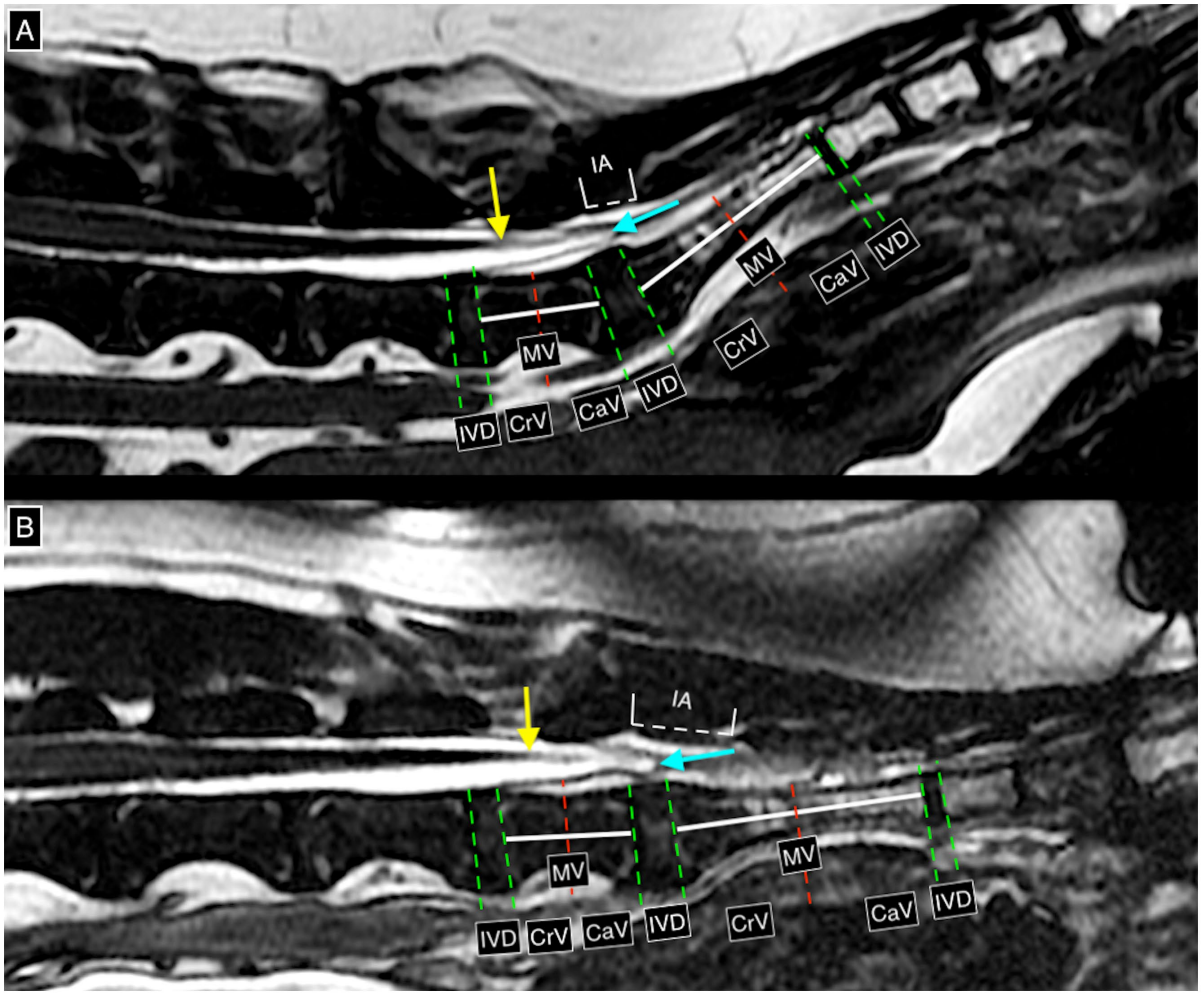
T2w 3D fast imaging employing steady-state acquisition (FIESTA) high resolution mid-sagittal images at the level of the caudal lumbar, sacral, and cranial coccygeal vertebral column and associated structures, on neutral **(A)** and flexed **(B)** lumbosacral angles (dynamic lumbosacral study), demonstrating the possible termination levels of the conus medullaris and dural sac. The red line is drawn perpendicularly in the middle of a white line connecting the mid-cranial and mid-caudal endplates of the same vertebra. The green lines are drawn over the margins of the vertebral endplates. In this case, the conus medullaris terminates at cranial L7 (yellow arrow) on both neutral and flexed lumbosacral angles (confirmed on transverse images). The dural sac terminates at the level of the intervertebral disc space L7-S1 (light blue arrow) in both neutral and flexed angles. IVD = intervertebral disc space, CrV = cranial vertebral body, MV = middle vertebral body, CaV = caudal vertebral body, IA = interarcuate space.

**Figure 2:**
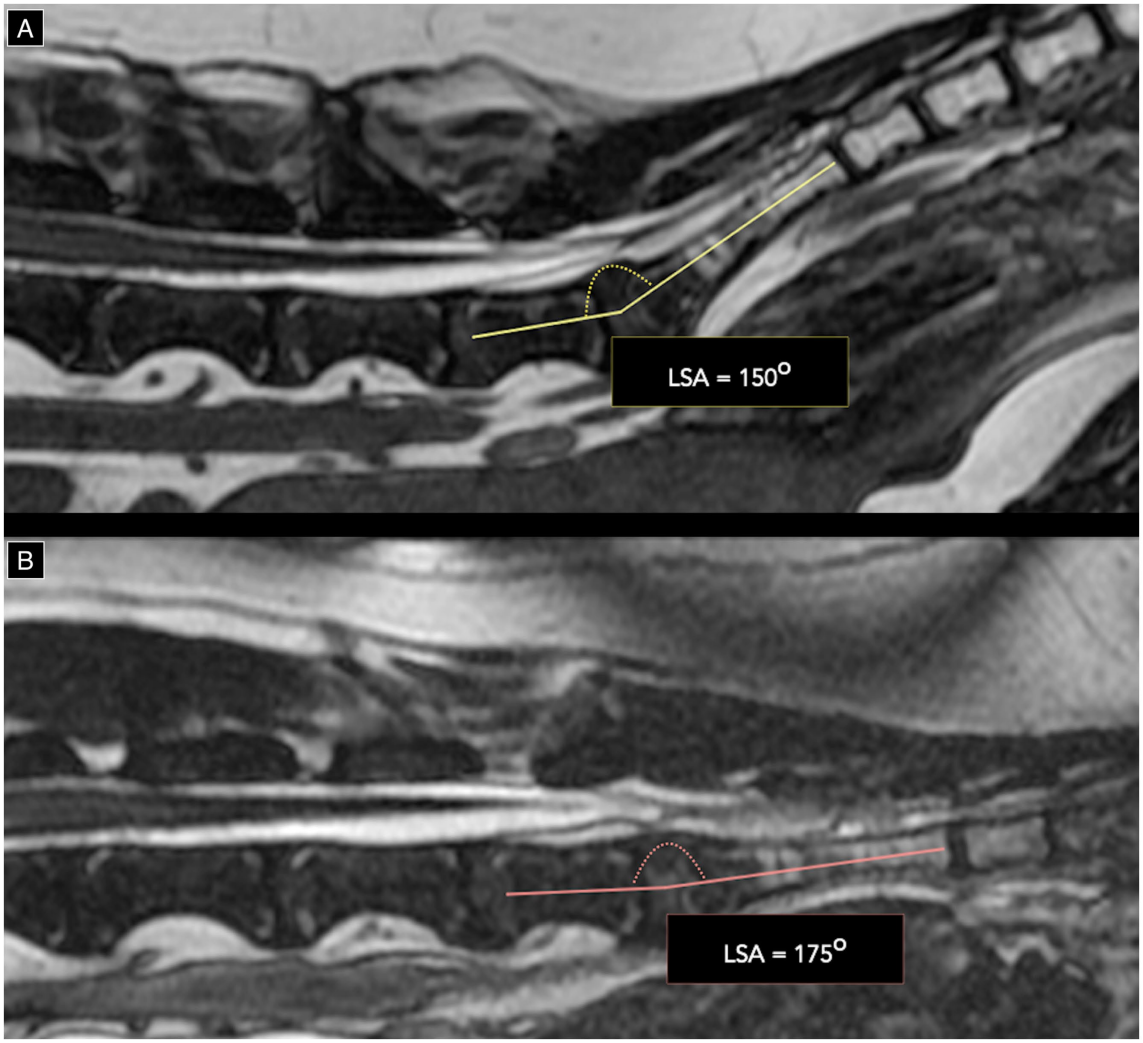
T2w 3D FIESTA high resolution mid-sagittal images of the lumbosacral junction on neutral **(A)** and flexed **(B)** position demonstrating a change in lumbosacral angle (LSA) from 150^o^ on neutral to 175^o^ on flexion.

The level of CMt and DSt was determined using methods adapted from previous studies (22, 23). Termination levels were categorized as a named intervertebral disc space or a cranial, middle, or caudal vertebral segment, as shown in Figure 1. Numerical values 1 to 11 were assigned to CMt and DSt, corresponding to entire vertebral body segments and intervertebral disc spaces from L5 to Cd1, to calculate the median level and range. Available dynamic lumbosacral MRI studies were evaluated for physiological cranio-caudal translocation of the CMt and DSt, as described in prior publications (18), with changes in angle noted as shown in Figure 2. Studies with no change in lumbosacral angle were considered non-dynamic. Cases with dynamic lumbosacral MRI were classified as having a fixed CMt and/or DSt if no cranio-caudal translocation was observed.

After establishing a clinical suspicion of OTCS and documenting unrelated concurrent diagnoses, management strategies were categorized as surgical, medical, or no treatment. Postoperative care for surgically treated dogs followed standard analgesic and anti-inflammatory protocols, and long-term pharmaceutical requirements were recorded at the last follow-up. Medical management was classified into polytherapy (two or more medications) or monotherapy (single medication). The use of non-steroidal anti-inflammatory drugs (NSAIDs), corticosteroids, gabapentinoids, and epidural corticosteroid injections was also documented. Intraoperative findings were assessed in all dogs that underwent surgical detethering, focusing on whether the FT*e*, FT*i*, or both were dissected. The choice of surgical target (FT*e*, FT*i*, or both) was based on the operating neurosurgeon’s clinical judgement, with supporting clinical reasoning not consistently recorded due to the retrospective nature of the study. Any additional intraoperative observations were documented separately.

Follow-up data were collected from clinical records, telephone updates, and communication with referring veterinarians, with video recordings reviewed when available. The follow-up duration was defined as the time from initial diagnosis to the last communication with the owner and/or referring veterinarian. Outcomes were classified as full recovery, partial recovery, no recovery/static, or deterioration. For C-OTCS dogs, short-term postoperative outcomes were assessed 2 months after surgery. Additionally, the number of dogs experiencing a relapse after initial recovery was recorded, with partial relapse defined as recurring signs without pre-treatment severity and full relapse as signs of equal or greater severity than before treatment. Finally, medical treatment response was evaluated for the entire cohort, including surgically managed cases prior to surgery.

### 2.4 Statistical analysis

Statistical analyses were conducted using R version 4.3.0 (R Foundation for Statistical Computing, Vienna, Austria) to examine associations between clinical and demographic variables and three key outcomes: response to medical therapy (responder = full/partial recovery, non-responder = static/deterioration), recovery status (full vs. no full recovery), and treatment status (on-treatment vs. off-treatment) at last follow-up. Treatment modality (medical vs. surgical) was included among the analyzed variables. Continuous variables were assessed for normality using the Shapiro–Wilk test. Normally distributed variables were summarized as mean ± standard deviation (SD) and compared using t-tests. Non-normally distributed variables were reported as median and interquartile range (IQR) and analyzed using the Mann–Whitney U test. Categorical variables were analyzed using chi-square tests, or Fisher’s exact tests when expected cell counts were <5. Receiver Operating Characteristic (ROC) curve analysis was performed for continuous variables with strong, statistically significant associations to determine optimal thresholds using Youden’s J statistic. The significance level was set at *p* < 0.05.

## 3 Results

### 3.1 Case recruitment

**Figure 3:**
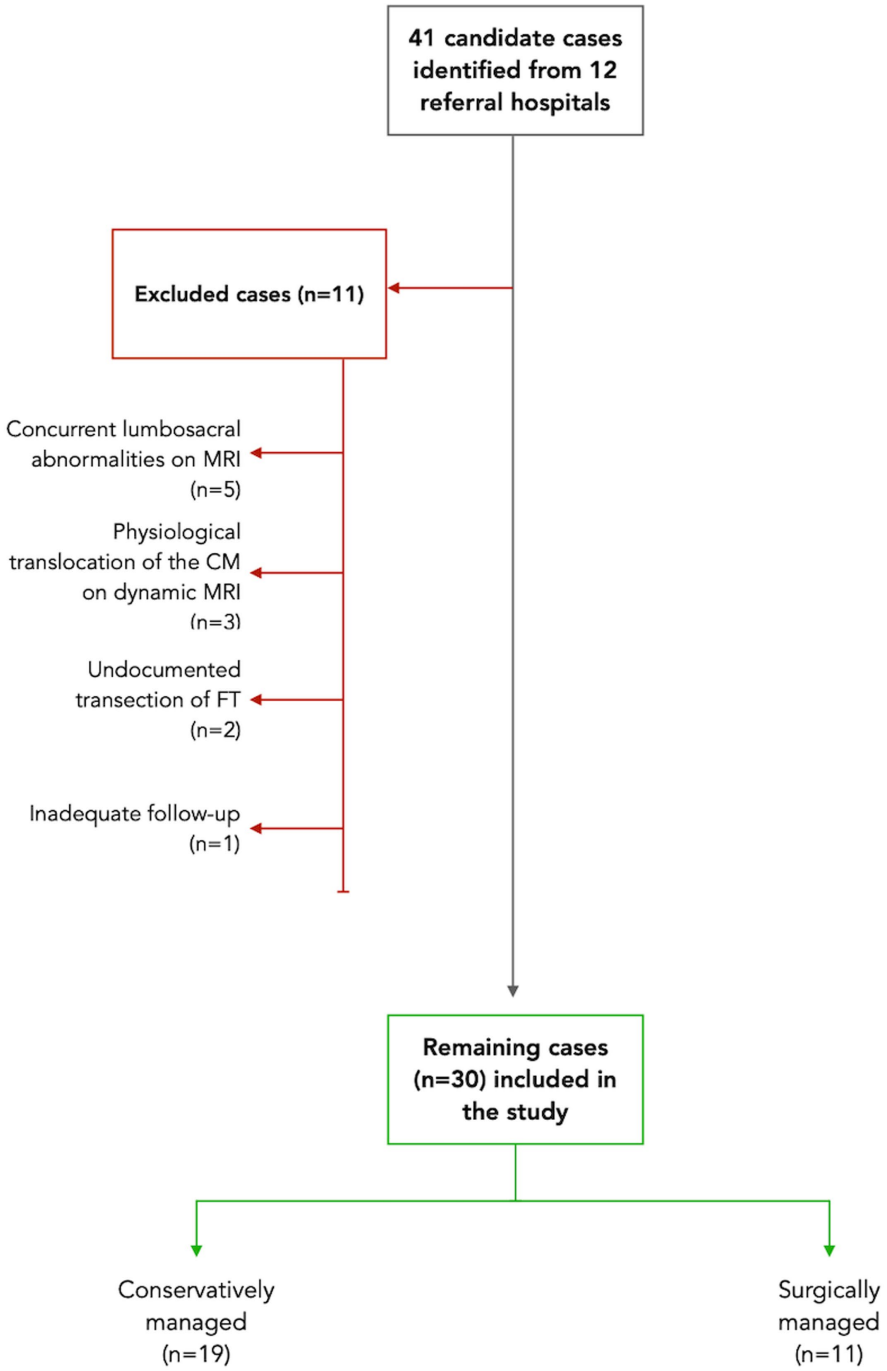
Study flow diagram illustrating case selection. A total of 41 candidate cases were initially identified from the clinical records of 12 referral hospitals based on the inclusion criteria. Eleven cases were excluded for various reasons: five had concurrent lumbosacral abnormalities on MRI, such as congenital malformations and degenerative changes; three conservatively managed cases showed conus medullaris (CM) physiological translocation on dynamic MRI; two surgically treated cases lacked documentation of transection of the filum terminale (FT) in the clinical notes; and one case had inadequate follow-up data. As a result, 30 cases met the study criteria: 19 were managed conservatively, while 11 underwent surgery.

The study selection process is illustrated in Figure 3. Nineteen conservatively managed dogs were classified as P-OTCS, while out of 11 dogs treated surgically all responded positively and were classified as C-OTCS. Included cases were presented between 2012 and 2025, and three had been reported in previous publications (14, 16, 17).

### 3.2 Signalment and clinical history

Demographic characteristics are presented in Table 3. Included breeds consisted of eight mixed-breed dogs, four Cocker Spaniels, four Cocker Spaniel–Poodle crosses, and one dog each from the following 14 breeds: Staffordshire Bull Terrier, Beagle, Weimaraner, Miniature Schnauzer, Braque du Bourbonnais, Shar Pei, Hungarian Vizsla, American Bulldog, Chihuahua, Havanese, Labradoodle (Labrador–Poodle cross), Spinone Italiano, Shiba Inu, and Rhodesian Ridgeback.

**Table 3:** Demographic and clinical characteristics in dogs with OTCS.

Diagnosis	No. of dogs	Sex				Age at onset in months Median (range)	Age at presentation in months Median (range)	Duration of clinical signs in months Median (range)	Body weight in kg Median (range)	Clinical severity score Median (range)
		Male		Female						
		Intact	Neutered	Intact	Neutered					
T-OTCS	30	13	9	5	3	11 (2–65)	25 (4–84)	13 (1–60)	15 (2–37)	5 (1–14)
C-OTCS	11	5	4	2	–	4 (2–65)	22 (4–71)	9 (1–44)	16 (4–23)	8 (3–14)
P-OTCS	19	8	5	3	3	18 (4–36)	28 (8–84)	14 (2–60)	11 (2–37)	4 (1–11)

T-OTCS, entire cohort of dogs with OTCS; C-OTCS, surgically managed confirmed OTCS group; P-OTCS, medically managed presumptive OTCS group.

Pain/dysesthesia involving the lumbosacral region, tail, and/or pelvic limbs was the most common presenting complaint reported in 29 dogs (97%; 11C-OTCS, 18 P-OTCS). Additional owner-reported clinical signs comprised gait abnormalities in 21 dogs (70%; 9C-OTCS, 12 P-OTCS), impaired physical activity in 19 (63%; 8C-OTCS, 11 P-OTCS), and urinary/fecal incontinence in five (17%; 4C-OTCS, 1 P-OTCS). Behavioral abnormalities were observed in 20 dogs (67%; 10C-OTCS, 10 P-OTCS) and are described in detail in Supplementary Table S1. Concurrent conditions were reported in 2 dogs consisting of gastritis and chronic enteropathy, respectively.

A deteriorating clinical evolution prior to referral was reported in 23 dogs (77%; 11C-OTCS, 12 P-OTCS). Six dogs (20%) were reported to be static, and one (3%) had improved, all with P-OTCS. All 11 dogs (100%) with C-OTCS and 16 dogs (84%) with P-OTCS had received medications for their condition. Out of these 27 dogs (90%), 11 had received monotherapy (41%), and 16 (59%) polytherapy, including non-steroidal anti-inflammatory drugs (NSAIDs) in 15 (56%), oral corticosteroids in two (7%), and gabapentinoids in 14 (52%). One dog had received two epidural infiltrations of methylprednisolone acetate (1 mg/kg) three weeks apart. Other medications included paracetamol (*n* = 5), phenobarbital (*n* = 2), propranolol (*n* = 1), acepromazine (*n* = 1) buprenorphine (*n* = 1), amantadine (*n* = 1), maropitant (*n* = 1) and flavoxate (*n* = 1).

### 3.3 Physical, orthopedic and neurological examination

No significant abnormalities were found on physical examination, except for self-inflicted skin lesions involving the pelvic limbs, scrotum, and tail of two dogs believed to be related to P-OTCS. All cases underwent orthopedic assessment. In C-OTCS, four dogs were reactive on hip extension. In P-OTCS, findings included grade I/II medial patellar luxation (*n* = 3), and pain on shoulder (*n* = 1) and hip extension with a skipping pelvic limb gait (*n* = 1). These were deemed unrelated to the presenting complaints based on orthopedic and neurological evaluations, supplemented by radiographs when necessary.

Details of all abnormalities identified on neurological examination are listed in Supplementary Table S1 and are grouped in clinical severity score categories in Supplementary Table S2. No dogs were reported to have additional neurological deficits localizing to neuroanatomical regions outside the L4-S3 spinal cord and/or associated spinal nerves/cauda equina. Clinical severity scores are summarized in Table 3. All dogs were graded neurologically based on the results of their clinical severity score, as presented in Table 2. Supplementary Videos 1, 2 demonstrate abnormal stiff, stilted pelvic limb gait and dysesthesia/neuropathic pain in the lumbosacral region/tail, respectively, in two dogs with C-OTCS.

### 3.4 MRI findings

A high-field MRI was performed in 27 dogs and a low-field in three. Four dogs underwent full head/brain studies, which showed no abnormalities. Twenty-eight studies were available for re-assessment. A dynamic lumbosacral MRI was performed in 20 dogs (67%; 8 C-OTCS, 12 P-OTCS), including 19 high-field and one low-field studies, all available for re-evaluation.

**Figure 4:**
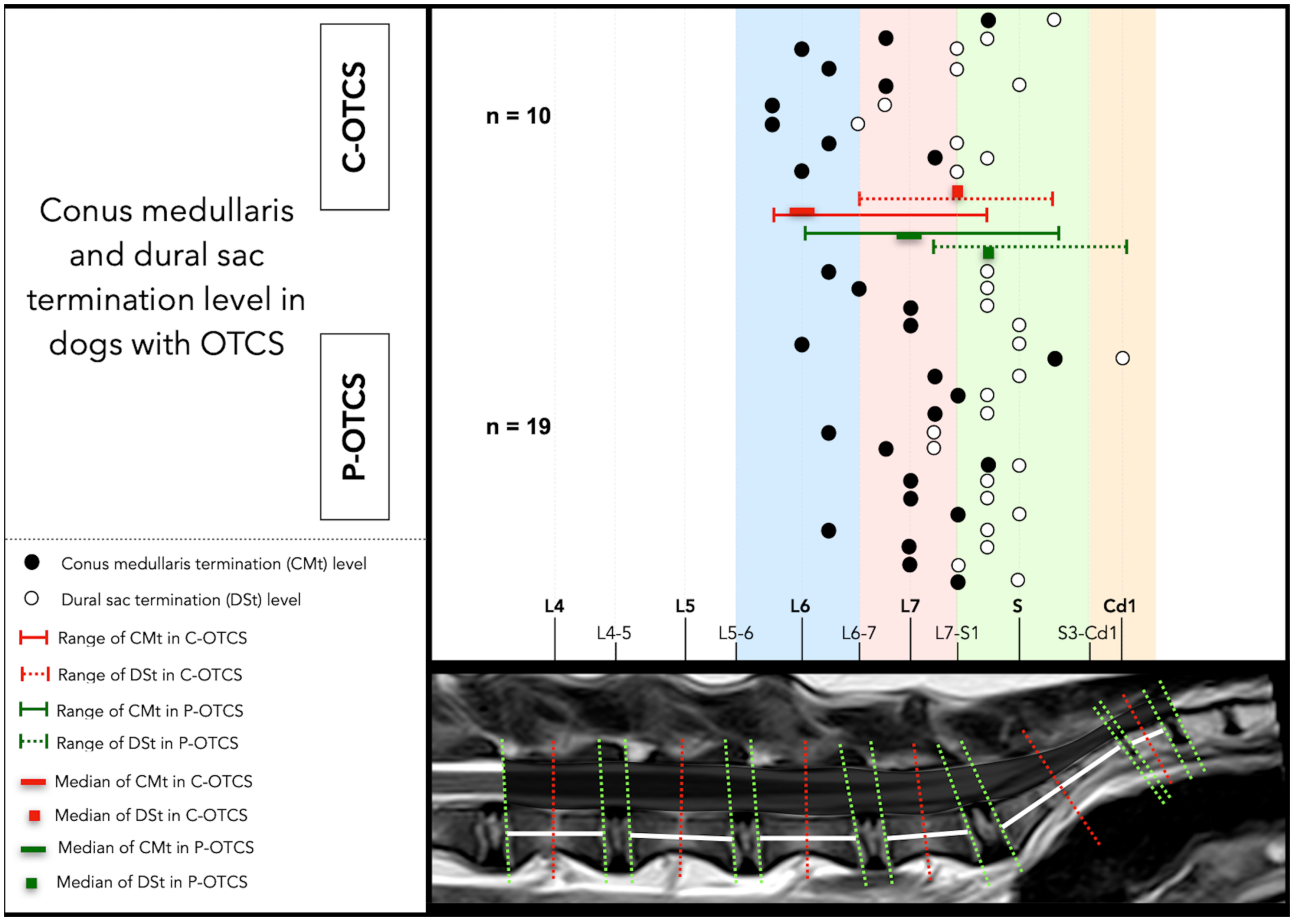
Anatomical distribution of conus medullaris and dural sac termination (CMt, DSt) levels in dogs with C-OTCS and P-OTCS. The plot illustrates individual termination levels for both structures, highlighting case-specific anatomical variations. The range and median termination levels for each group are also shown.

Individual CMt and DSt in neutral position, alongside the range and median in each group were determined in 29 cases (Figure 4). One case, imaged with low-field MRI, was excluded from analysis due to lack of agreement. In the entire cohort, the median and most common CMt was at L7 (*n* = 7), while the median and most common DSt was at S1/cranial sacrum (*n* = 11).

Among 20 dynamic lumbosacral MRI studies, only one (the sole low-field dynamic study, diagnosed with C-OTCS) was of insufficient quality to assess CM or DS translocation. In the remaining 19 dogs, sagittal, with or without transverse and/or dorsal images, were acquired in both extension and flexion in 12 cases, in flexion alone in five, and in extension alone in two. The median angle was 153° in the neutral position across 27 MRI studies, 178° in flexed position across 17 studies, and 144° in extended position across 14 studies. None of the cases with dynamic lumbosacral MRI demonstrated any appreciable cranial (with extension) or caudal (with flexion) translocation of the CMt or DSt and were therefore classified as having a fixed position.

### 3.5 Other investigations

Complete blood count and serum biochemistry were performed in 27 and 28 dogs, respectively, with unremarkable results. CSF PCR for *Toxoplasma gondii* and *Neospora caninum* (*n* = 1), serum *T. gondii* IgM/IgG (*n* = 2), *N. caninum* serology (*n* = 2), phenobarbital serum concentration (*n* = 1), adrenocorticotropic hormone stimulation (*n* = 1), low-dose dexamethasone suppression test (*n* = 1), and cytology from a jejunal lymph node (*n* = 1) all yielded unremarkable results. CSF analysis was performed in 13 cases (11 lumbar, 2 unspecified), with no abnormalities detected.

Electrodiagnostic investigations were performed in nine cases (30%), with abnormalities detected in four (44%): three with P-OTCS and one with C-OTCS. All three dogs with P-OTCS and electrophysiological abnormalities exhibited a fixed CM position on dynamic lumbosacral MRI. Evaluated regions included the pelvic limbs, tail base, sacral, lumbar, thoracic, cervical muscles, and thoracic limbs. Tests included electromyography (EMG) in all, motor nerve conduction (MNC) in six, repetitive nerve stimulation (RNS) in five, sensory nerve conduction (SNC) in four, cord dorsum potential (CDP) in one, and F-wave study in one. Electromyographic abnormalities included fibrillation potentials and positive sharp waves at the tail base (*n* = 4) and proximal pelvic limbs (*n* = 1). One dog had mildly reduced MNC velocity in the right sciatic-tibial nerve (45 m/s). Another showed polyphasic F-waves (right tibial nerve, hock stimulation) with increased minimum F wave latency (28.1 m/s, expected = 16.65 m/s) and an F-ratio of 4.5 (normal = 1.75 ± 0.02). At hock stimulation of the left tibial nerve, F-waves showed increased latency (22.2 m/s) and an F-ratio of 3.3.

Additional diagnostic imaging procedures were unremarkable and included: radiography of the pelvis (*n* = 2), coxofemoral joints (*n* = 2), thoracolumbar (*n* = 1) and lumbosacral vertebral column (*n* = 1), shoulders (*n* = 1), and stifles (*n* = 1); computed tomography of the lumbosacral vertebral column (*n* = 2), abdomen (*n* = 1), and pelvic limbs (*n* = 1); abdominal (*n* = 3) and lumbar epaxial musculature (*n* = 1) ultrasonography, endoscopy (*n* = 1), and cystography (*n* = 1).

Histopathology was performed in six of 11 dogs undergoing surgery (C-OTCS) and confirmed the origin of the dissected tissue as FT. Histopathological descriptions were consistent across cases and included: (a) mature, well-differentiated, cell-poor, abundant, collagenous stroma of dense fibrous connective tissue, with elastin and collagen in parallel arrangement; (b) central, palisading, tubular-type, columnar cells with rare visible cilia consistent with ependyma and extension of the central canal; (c) occasional capillaries in the periphery, bundles of myelinated axons, and rare neutrophils.

### 3.6 Treatment

Seventeen dogs (57%) received medical treatment, two remained untreated post-diagnosis, and 11 (37%) underwent surgery after failing medical management. All surgically treated dogs received polytherapy with peri- and postoperative tapering analgesic protocols, including NSAIDs (*n* = 7), gabapentin (*n* = 10), paracetamol (*n* = 3), amitriptyline (*n* = 3), amantadine (*n* = 3), prednisolone (*n* = 1), and pregabalin (*n* = 1); one received flavoxate. Among P-OTCS cases, seven of 17 received monotherapy, and 10 polytherapy, using NSAIDs (*n* = 3), corticosteroids (*n* = 2), gabapentin (*n* = 13), paracetamol (*n* = 5), fluoxetine (*n* = 1), amantadine (*n* = 4), alprazolam (*n* = 1), and amitriptyline (*n* = 1). One dog received three epidural methylprednisolone (1 mg/kg) injections.

**Figure 5:**
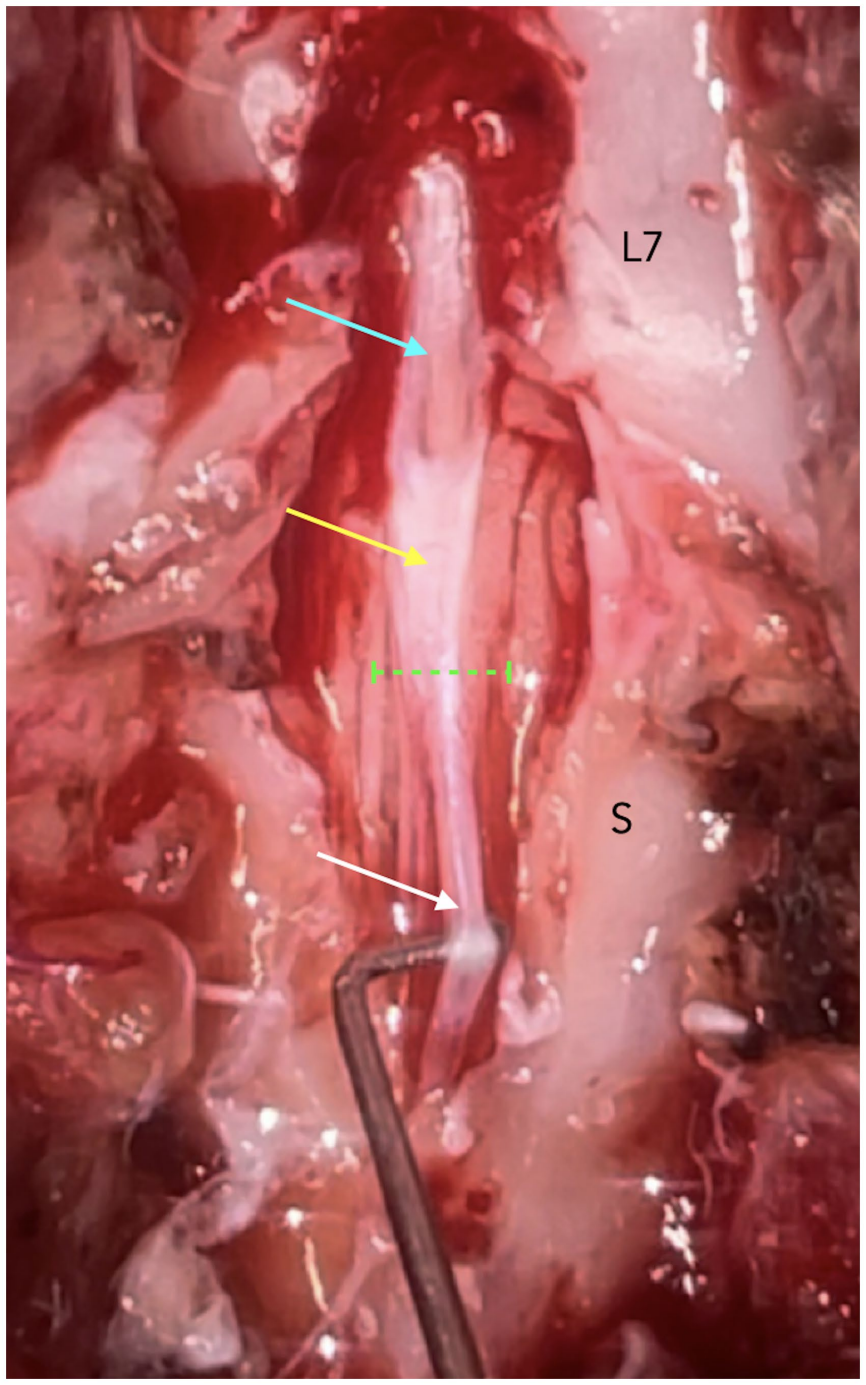
Dorsal laminectomy from L7 to S2 in a dog with C-OTCS showing normal dural sac (cyan arrow), thickened and more opaque dura (yellow arrow) near the termination of the dural sac (interrupted green line), and the filum terminale externum (white arrow).

**Figure 6:**
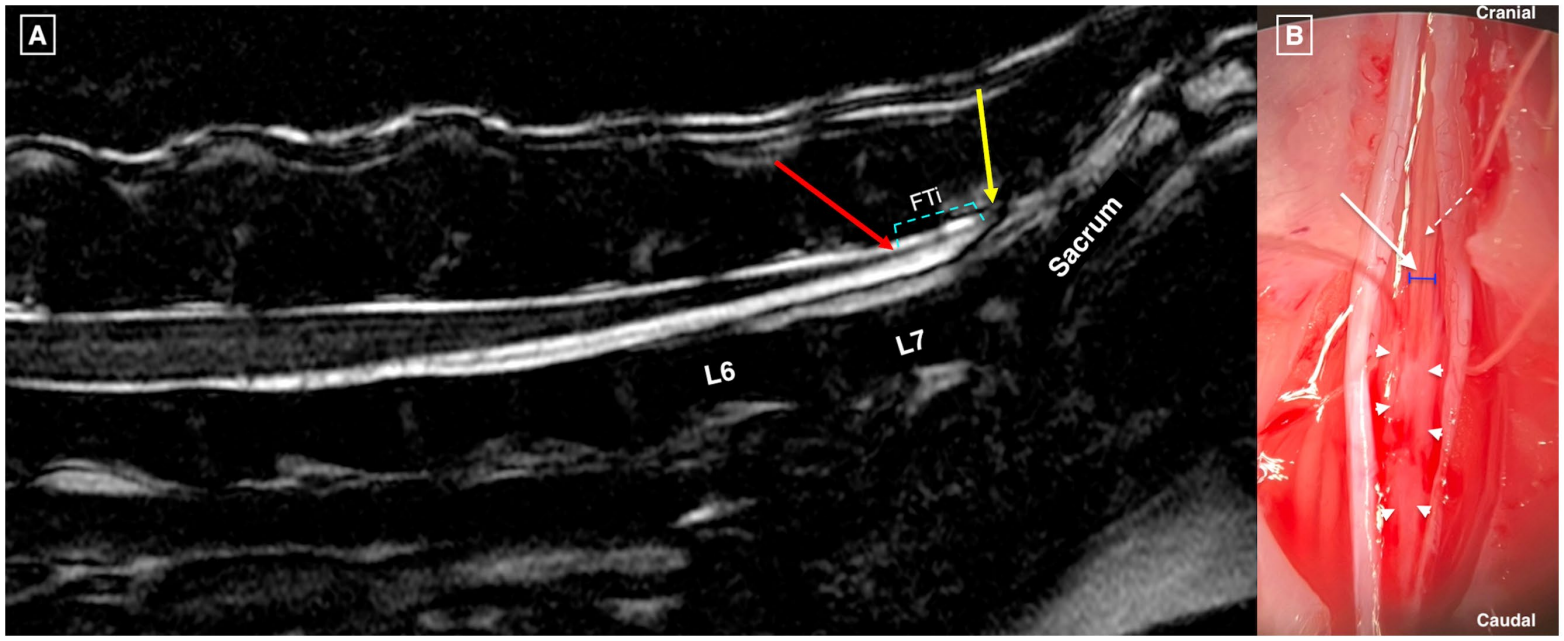
T2w 3D FIESTA high resolution mid-sagittal image of the caudal lumbar, sacral, and cranial coccygeal vertebral column in a dog with OTCS showing no visible structural abnormalities **(A)**. The conus medullaris terminates at mid-L7 (red arrow), the dural sac terminates at the cranial sacrum (yellow arrow), and the filum terminale internum (FT*i*) is marked with cyan interrupted lines. Panel **(B)**: Dorsal microsurgical view after dorsal laminectomy at L7-S2 in the same dog. The dural edges are retracted after durotomy, revealing the FT*i* (white arrow), its width (blue lines), and the dorsal spinal vein of the filum terminale (interrupted white arrow). Abnormal infiltrative soft tissue (white to gray) adheres to the distal FT*i* (white arrowheads) and extends caudally to the termination of the dural sac.

**Figure 7:**
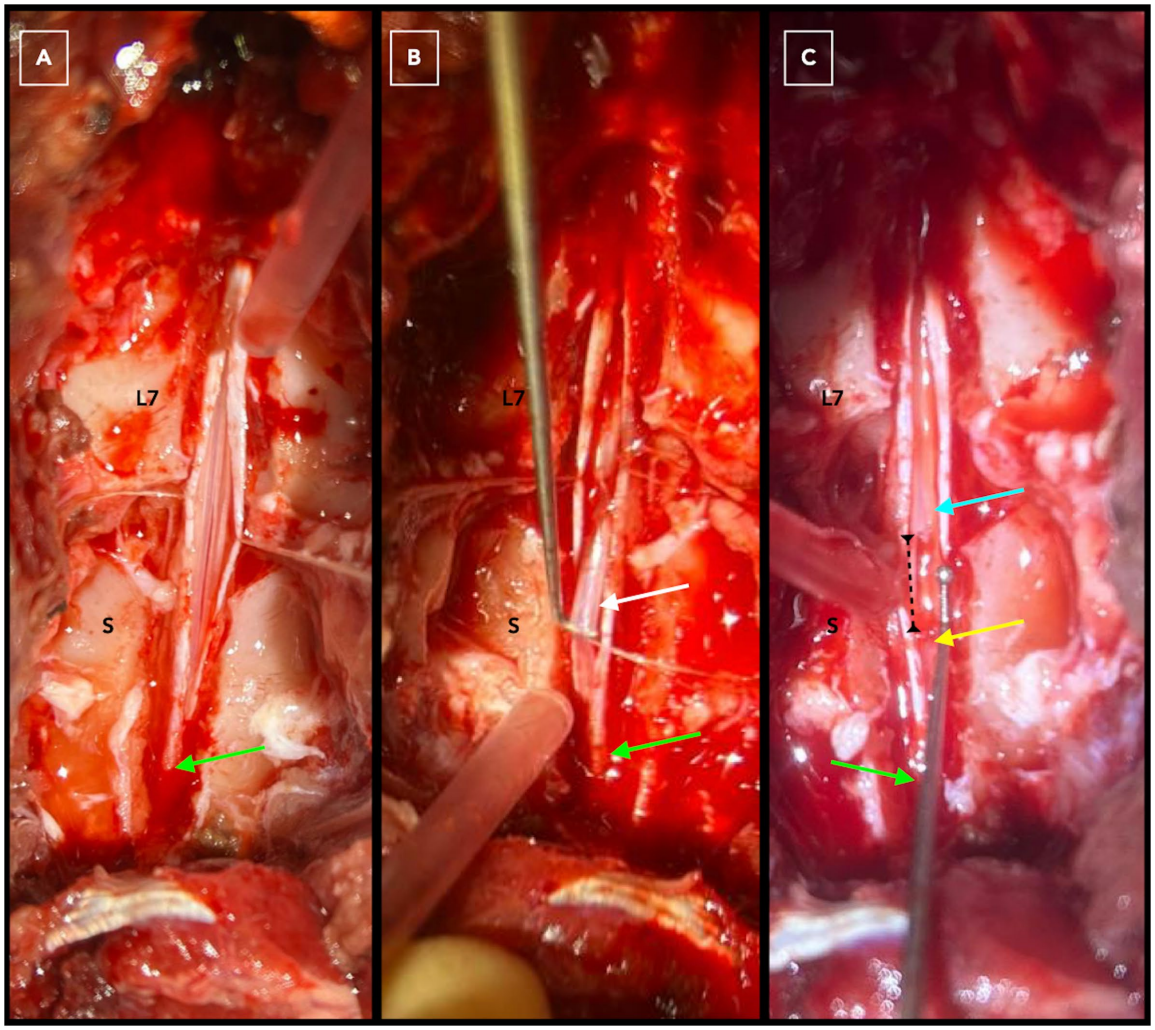
Dorsal laminectomy at L7-S3 in a dog with C-OTCS followed by durotomy from the cranial L7 vertebra to the termination of the dural sac in the sacrum (green arrow) for exposure of intradural structures **(A)**. The filum terminale internum is highlighted by the white arrow before **(B)** and after **(C)** dissection. The cyan arrow marks the proximal segment of the filum terminale internum, while the yellow arrow points to the distal remaining part. The interrupted black line indicates the distance created after dissection and cranial translocation of the conus medullaris.

A lumbosacral dorsal laminectomy was performed in C-OTCS cases, with or without modifications: L7-S1 (*n* = 4), L7-S2 (*n* = 4), L7-S3 (*n* = 1), L7-Cd1 (*n* = 1), and S1-S2 (*n* = 1). Surgical positioning adhered to standard protocols, with the dog placed in sternal recumbency, the pelvic limbs either flexed forward or in a neutral frog-leg posture, and support provided beneath the pubis. Surgery involved dissection of the FT*e* alone in five cases and both FT*e* and FT*i* in six. A thickened FT was subjectively noted in eight dogs (73%), a tight FT in nine (82%), and cranial translocation of the DS/CM after FT dissection in eight (73%). One dog had a thickened DSt with infiltrative fibrotic tissue adhered to the FT*i*. Biosynthetic (*n* = 3; Vetrix BioSIS, Cumming, GA, USA; Lyoplant, B Braun Medical Ltd., Sheffield, UK) or fat (*n* = 1) grafts were placed over the laminectomy and/or durotomy window in four cases. A titanium mesh implant (Veterinary Instrumentation, Sheffield, UK) was secured with 1.5×5 mm self-tapping titanium screws, one over L7 and another over the sacrum in one case. The FT*e* is shown in Supplementary Video 3 and Figure 5, while the FT*i* is visualized in Figures 6, 7. No intra- or postoperative complications or neurological deterioration were reported.

### 3.7 Follow-up and outcome

The median follow-up after diagnosis was 9 months (range: 2–108; 15 in C-OTCS, 6 in P-OTCS). All but two dogs were re-examined by a board-certified/board-eligible neurologist at a median of 4 months post-diagnosis (range: 1–20). The remaining two were assessed by the referring veterinary surgeon.

In C-OTCS, short-term outcomes showed full recovery in 9/11 dogs (82%) and partial recovery in 2/11 (18%). At the last follow-up, full recovery declined to 7/11 (64%), as two dogs experienced partial relapses at 6 months postoperatively and were reclassified as partial recovery. Relapsing signs included episodic lumbosacral pain/dysesthesia and reduced physical activity, not reaching pre-treatment severity. One additional dog with partial recovery exhibited relapsing lumbosacral pain/dysesthesia 1 month postoperatively. Two of these three relapsed cases underwent repeat surgery: one was re-operated 11 days and the other 6 months after repeated imaging. Both dogs showed notable but incomplete improvement, with significant clinical gains evident from the early postoperative period. The overall clinical outcomes at the last follow-up are summarized in Table 4. The same dog shown in Supplementary Video 1 can be seen in Supplementary Video 4, 8 days post-operatively, demonstrating remarkable improvement of previous gait abnormalities despite tapering medical therapy.

**Table 4:** Overall clinical outcome at last follow-up in dogs with OTCS.

Clinical evolution (outcome)	T-OTCS No. of dogs (%)	C-OTCS No. of dogs (%)	P-OTCS No. of dogs (%)
Full recovery	10 (37)	7 (64)	3 (16)
Partial recovery	13 (43)	4 (36)	9 (47)
Static	6 (20)	0 (0)	6 (16)
Deterioration	1 (3)	0 (0)	1* (5)

T-OTCS, entire cohort of dogs with OTCS; C-OTCS, surgically managed confirmed OTCS group; P-OTCS, medically managed presumptive OTCS group.

^*^Euthanasia was elected because of poor quality of life related to P-OTCS.

All three C-OTCS dogs with relapse underwent repeat MRI, revealing: (a) mild dorsal displacement of the DS beyond the laminectomy window, with absent cranio-caudal translocation of the CM on dynamic imaging; (b) cranial positioning of the CM compared to initial MRI, with absent cranio-caudal translocation and presumed fibrotic soft tissue bulging into the laminectomy window; (c) dorsal displacement of the cauda equina in contact with contrast-enhancing soft tissue. Two dogs underwent revision surgery involving DS and cauda equina detethering via excision of fibrotic tissue and adhered fat (Supplementary Video 5). One also required durectomy (Supplementary Video 6), dissection of intra-dural adhesions, and excision of the FT*i*. Histopathology confirmed correct tissue origin and revealed pachymeningitis with lymphoplasmacytic and neutrophilic inflammation, chronic hemorrhage, suspected ependymal cells, and dense collagen deposition. Further intervention was declined for the third dog, and MRI findings remained unverified surgically.

At last follow-up, 17 dogs (57%) remained on medical treatment: three (27%) in C-OTCS (2 polytherapy, 1 monotherapy) and 14 (74%) in P-OTCS (9 monotherapy, 5 polytherapy). Treatment had been discontinued in ten dogs, and two dogs had never received medical treatment, having been managed with exercise adjustments only. One dog had been euthanized due to worsening pain/dysesthesia and aggression.

Two medically managed dogs underwent microsurgical detethering prior to manuscript submission and showed positive response but were excluded from C-OTCS analysis due to insufficient post-operative follow-up. Intraoperatively, the FT*i* was found embedded in suspected fibrotic adhesions (Figure 6), and the DSt was thickened and opaque. Histopathology confirmed dense mature fibrous connective tissue in both cases, with focal fibrosis suspected in one and not excluded in the other.

### 3.8 Prognostic factors

**Figure 8:**
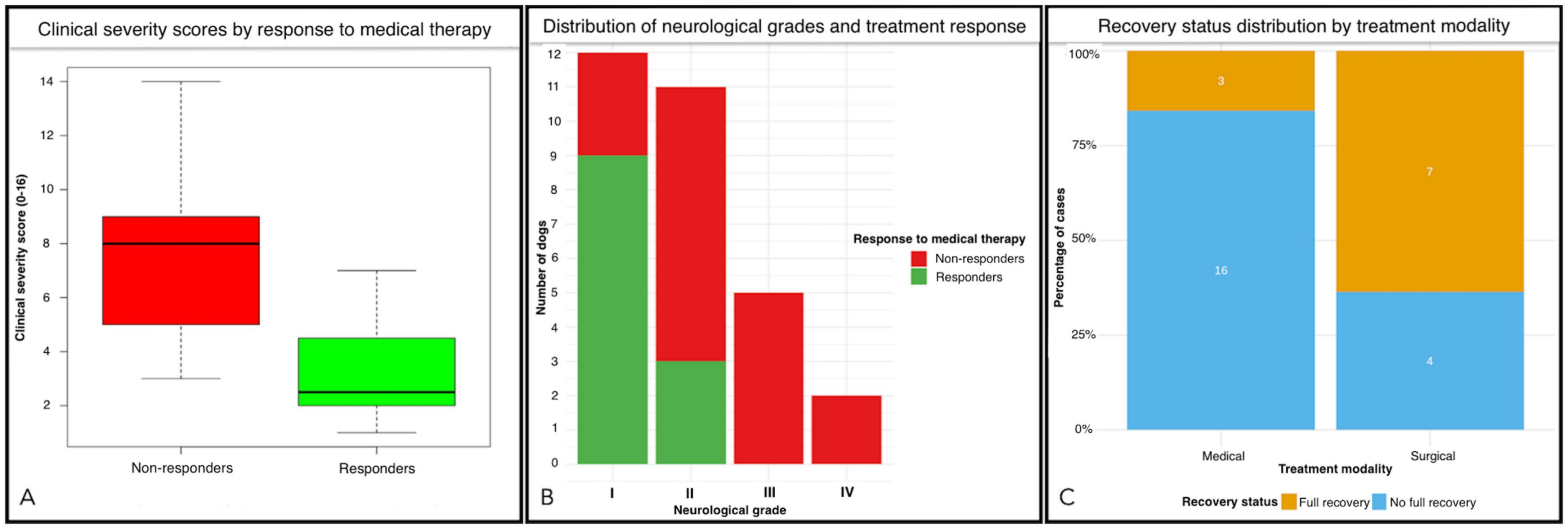
Panel **(A)**: Boxplot comparing clinical severity scores between responders and non-responders to medical therapy in dogs with OTCS. Responders exhibited significantly lower clinical severity scores (mean = 3.25) compared to non-responders (mean = 7.78), with *p* < 0.001. In panel **(B)**, a stacked bar chart showing the relationship between neurological grades (I-IV) and medical treatment response can be seen. Grade I patients had the highest response rate (75%, 9/12), followed by Grade II (27%, 3/11), with no response observed in Grade III (0/5) or Grade IV (0/2) patients. The chart highlights a significant association between lower neurological grades and positive medical treatment response (*p* = 0.006). In panel **(C)**, the proportion of recovery outcomes (full recovery vs. no full recovery) at last follow-up, stratified by treatment modality (surgical vs. medical) is also shown. Surgical treatment is associated with a significantly higher proportion of full recovery compared to medical treatment (*p* = 0.015).

Clinical severity scores strongly predicted medical treatment response (responders: 3.25 ± 2.09 vs. non-responders: 7.78 ± 3.15; Figure 8), with lower scores significantly associated with positive response (*p* < 0.001). ROC analysis confirmed its discriminative value (AUC = 0.90, 95% CI: 0.79–1.00) with an optimal threshold of 7 (sensitivity: 100%, specificity: 61%). Deteriorating clinical evolution prior to referral (*p* = 0.009), presence of behavioral abnormalities (*p* = 0.045), and higher neurological grades (*p* = 0.006; Figure 8) were negatively associated with positive response to medical therapy. Grade I had the highest response rate (75%, 9/12), followed by Grade II (27%, 3/11), with no response observed in Grade III (0/5) or Grade IV (0/2). Surgical treatment was significantly associated with full recovery (*p* = 0.015; Figure 8) and treatment discontinuation at last follow-up (*p* = 0.023).

No associations were found between medical treatment response, recovery or treatment status, and variables including age at onset, age at presentation, duration of clinical signs, body weight, sex, neuter status, gait abnormalities, pain/dysesthesia, urinary/fecal incontinence, neurological deficits, or physical activity impairment at presentation (*p* > 0.05). Additionally, behavioral abnormalities, clinical severity score and neurological grade were not associated with recovery or treatment status (*p* > 0.05).

In surgically managed dogs (*n* = 11), those achieving full recovery had lower clinical severity scores (median: 6.0, range: 3–9) than those without full recovery (median: 11.5, range: 8–14). Dogs without relapse or ongoing treatment had lower scores (median: 7.0, range: 3–10) than those with relapse or requiring treatment (median: 13.0, range: 8–14). All six dogs undergoing combined FT*i* and FT*e* surgery fully recovered without relapse or ongoing treatment, whereas only one of five undergoing FT*e* excision alone fully recovered, and three relapsed. Statistical analysis was not performed in this subgroup due to small sample size.

## 4 Discussion

Occult tethered cord syndrome is rarely reported and poorly characterized in dogs but can lead to debilitating clinical signs (16, 17). Owing to the absence of abnormalities on conventional MRI and undefined clinical presentation, this enigmatic disorder is likely underdiagnosed (28). As a treatable condition with positive outcomes in both humans and dogs (16, 17, 29), understanding its clinical phenotype is crucial for accurate identification and management. This study is the first to describe the clinical signs, MRI findings, treatment, and outcomes in dogs with surgically treated confirmed or medically managed presumptive OTCS, while also introducing novel prognostic factors. Advances in imaging criteria (19) and the identification of prognostic indicators (21, 29–31) have improved understanding of OTCS in humans, though the diagnostic process remains difficult (30).

A key characteristic of the typical presentation of OTCS in our cohort was the juvenile clinical onset (32), particularly in dogs with C-OTCS. In human medicine, the mean age at surgery has been reported as 16 years in one study (29) and 24 in another (21). A prolonged reported duration of clinical signs, ranging from several months to over 10 years, points to an even younger age at onset. These findings align with our results, further supporting the hypothesis that OTCS may be congenital (33), and underscoring the importance of signalment in diagnosing this condition. However, the onset of clinical signs as late as 65 months in one C-OTCS dog suggests a possible acquired etiology, while presentation between 12 and 36 months in others raises the possibility of subclinical disease, where early signs may go unrecognized.

In humans with OTCS, the main clinical signs are pain (70%), neurological deficits (74%), and loss of bladder or bowel control (over 60%), collectively forming the tethered cord syndrome symptom triad (21, 34). Similarly, pain was the most common sign in our cohort, followed by neurological deficits, though urinary/fecal incontinence was less frequent. This may reflect a true interspecies variation or overlooked subclinical signs such as urgency to urinate/defecate. Moreover, urodynamic studies, commonly used in humans to assess neuropathic bladder dysfunction (35), were not performed in our dogs. Notably, 23% of the cohort exhibited pain during defecation/urination, which may represent a distinctive sign of OTCS in the absence of alternative diagnoses. Perineal pain and painful urination are documented in humans (36), and this further supports that, despite certain differences, a similar clinical profile may be shared between human and canine OTCS.

Behavioral abnormalities were frequently observed in our cohort. These included increased anxiety, restlessness, defensive aggression or vocalization triggered by touch, self-mutilation, excessive limb licking, and compulsive behaviors, all directed at the lumbar/lumbosacral region, tail, and/or pelvic limbs. The resolution or notable improvement of these signs following surgery suggests a pain/dysesthesia-related origin. Similar manifestations were reported in military working dogs with multilevel lumbosacral stenosis (27), further supporting the impact of chronic pain on quality of life and its role in triggering various behavioral responses in dogs (37, 38). It induces emotional distress but also neurobiological changes such as central sensitization, hyperesthesia, allodynia, and the wind-up phenomenon, with cumulative effects over time (37). Based on our results, dogs with OTCS are subjected to chronic pain, which likely predisposes them to persistent and potentially ingrained behavioral patterns that may resist medical therapy. Thus, early recognition of pain-related behaviors is essential for improving patient welfare and treatment outcomes.

Diagnosing OTCS is inherently challenging. In this cohort, surgical detethering via FT dissection resulted in resolution or remarkable improvement of previously pharmacoresistant clinical signs, reinforcing diagnostic validity. Clinical suspicion was based on age at clinical onset, persisting/deteriorating signs, anatomical neuro-localization with strict exclusion of alternative diagnoses, and the identification of a fixed CM in dynamic MRI studies, when available. Notably, no cases with post-operative findings contradicting the initial OTCS diagnosis were identified during recruitment, further validating the diagnostic approach and clinical reasoning behind surgical intervention. Despite these findings, OTCS remains a contentious condition with a challenging pre-surgical diagnostic process, especially given the reliance on clinical suspicion in the face of normal static (non-dynamic) MRI results. This is underscored by the prolonged duration of clinical signs, numerous diagnostic tests, and repeated failures to reach a diagnosis prior to referral. These challenges highlight the urgent need for robust diagnostic criteria that guide clinical decision-making.

A significant correlation was found between our novel 16-point clinical severity scoring system and response to medical treatment, aligning with the 15-item scale used in human OTCS (21). Higher scores were associated with reduced likelihood of response to medical therapy, supporting the system’s clinical relevance. ROC analysis confirmed strong discriminative power, suggesting its utility in identifying dogs more likely to benefit from medical management, though some non-responders may be misclassified. Stratification in neurological grades simplified clinical interpretation, showing that most grade I dogs responded favorably, whereas higher grades correlated with poorer outcomes. In surgically managed dogs, lower severity scores were observed in those achieving full recovery, without relapse or need for ongoing treatment, suggesting an additional potential to predict surgical outcomes. However, these findings were purely descriptive, with C-OTCS sample size precluding statistical verification. The proposed clinical severity scoring system should only be applied to dogs meeting the study’s inclusion criteria, with alternative diagnoses ruled out. Moreover, our results should be interpreted with caution, and further validation with a larger cohort is needed to confirm their robustness and clinical utility in guiding treatment decisions in canine OTCS.

The pathophysiology of canine OTCS remains unclear. In humans, FT microstructural abnormalities are suspected (12, 13, 33, 34), and similar mechanisms are proposed in dogs (14, 16, 17). The FT consists of the FT*i*, extending from the CMt to the DSt, and the FT*e*, anchoring the DSt to osseous structures. It stabilizes the spinal cord, buffers traction, and allows limited sliding (12, 13, 39, 40). In dogs, detailed anatomical descriptions of the normal FT are lacking, impeding our understanding of potential deviations that may contribute to the development of OTCS. A recent human cadaveric study has provided comprehensive data on the morphological and morphometric characteristics of the FT, highlighting a variable distal insertion with strand-like structures enveloped in vascular tissue (15). Histologically, the FT contains collagen, elastic fibers, adipose tissue, and neural elements, including ependyma, glial cells, axons, and dorsal root ganglia (41–43). In our study, neural tissue and a persistent central canal were also observed, aligning with the known cellular composition of the FT in humans, and should not be regarded as pathological. These neural elements are embryologic remnants from secondary neurulation, the process that also forms the cauda equina and CM (38, 40, 41). While deficits in this process could lead to OTCS/TCS (34, 44), the exact mechanisms remain unknown. Mechanoreceptors and nociceptors in the FT further support its role in OTCS pathophysiology (42). Pathological FT exhibits dense collagen, hyaline degeneration, reduced elasticity, and vascular changes leading to hypoxia and tissue damage (11, 13, 34, 45). Our histopathological analysis revealed dense fibrous tissue in the FT, which was not classified as abnormal due to the absence of defined histopathological criteria for canine OTCS. To advance our understanding of OTCS, standardized anatomical and histological investigations that establish baseline reference data are crucial in veterinary medicine to identify pathological structural deviations in the canine FT as potential biomarkers of the condition.

Conventional MRI is limited in diagnosing OTCS, with the term ‘occult’ referring to a radiologically normal FT despite microstructural and functional defects (9–11). This was also demonstrated in some of our cases where no abnormalities were seen on MRI, but suspected fibrotic infiltrative tissue was identified intra-operatively embedding the FT*i*. This lack of distinct MRI findings complicates the diagnosis and can delay surgical intervention. The advent of dynamic lumbosacral MRI has marked a significant advancement, identifying cases with a fixed CM, and increasing clinical suspicion in the absence of alternative diagnoses (14). In dogs, the CM exhibits physiological cranial and caudal movement during flexion and extension, respectively (18). Conus medullaris translocation is greater during flexion (9 mm) compared to extension (−2.5 mm) and is strongly associated with the change in lumbosacral angle (18). In human medicine, the absence of movement during dynamic MRI is characteristic of OTCS, contrasting with the physiological translocation seen in healthy subjects (19, 46). While the accuracy of dynamic MRI in dogs requires further validation, our results support its diagnostic utility. Additionally, the similarity of our measured lumbosacral angles in neutral, flexed, and extended positions to previously reported values (18) reinforces the procedural validity of this imaging technique.

Recent studies in human medicine have explored complementary diagnostic tools. Ultrasound elastography, assessing shear wave velocity (SWV), has shown promise as a non-invasive measure of spinal cord tension, validated in simulated, cadaveric, and intraoperative settings (47). Decreased SWV is observed following neurosurgical interventions for TCS, suggesting its potential as an adjunct to traditional imaging (47). *In vivo* SWE studies in dogs have demonstrated that spinal cord compression increases stiffness, providing biomechanical insights with potential applicability in veterinary patients (48). Diffusion tensor imaging (DTI) has also proven useful for detecting spinal cord damage in TCS, revealing changes in tissue properties that could inform surgical decisions (49). The clinical application of these techniques in veterinary medicine warrants further investigation to improve diagnostic confidence in conditions that are inherently difficult to diagnose, such as OTCS.

The terminal position of the CM in dogs varies, influenced by body weight, while the DSt is typically at the cranial sacrum (22). In our cohort, both CMt and DSt showed greater variability than previously reported, though the most frequent termination sites aligned with established anatomical configurations (22), complicating conventional TCS diagnosis. This parallels human OTCS, where the CM is normally positioned, while a “low-lying” CM is indicative of conventional TCS (11). In dogs, identifying a pathologically “low-lying” CM is challenging due to breed-related variations, as seen in Cavalier King Charles Spaniels (CKCS), where both the CMt and DSt are naturally more caudal (23). Thus, some of our cases may represent conventional TCS rather than OTCS. The radiologic characteristics of the FT represent another important diagnostic consideration. Pain without syringomyelia in CKCS correlates with a shorter FT*i* (50), suggesting FT*i* length may offer diagnostic value, though it was not assessed in this study. Similarly, FT thickness is measured in human OTCS (51), but remains technically challenging in dogs, requiring high-resolution, artifact-free, thin-slice MRI— a limitation in some cases of this cohort, reflecting challenges in clinical application. Future studies should establish normal FT morphology on MRI to enhance OTCS diagnosis.

Electrodiagnostic investigations revealed non-specific abnormalities. An overlap between TCS-related signs and neuromuscular disorders has been documented in humans (52), and some signs in our cohort were exacerbated by exercise, prompting electrodiagnostic evaluation. In human TCS, reported abnormalities include tibial somatosensory evoked potential cortical response changes (60%), chronic neurogenic EMG activity (over 40%), reduced motor unit action potential amplitudes, and abnormal H reflexes. These findings suggest sensory and motor pathway dysfunction due to chronic traction and ischemia affecting the spinal cord and nerve roots (53). Electrodiagnostics may also aid intraoperative neurophysiological monitoring (IONM) by helping localize the FT and differentiate it from nerve roots, enhancing surgical safety (54, 55). However, IONM is ineffective for a normal FT, as it responds similarly to the cauda equina under electrical stimulation (56). Moreover, IONM use in veterinary practice is limited by impracticality, unavailability, and added costs. In the author’s experience, identifying the FT*i* and associated pathology in small and miniature breeds is particularly challenging, with surgical microscopy being necessary and more accessible in specialist clinical practice. In conclusion, electrodiagnostic findings in canine OTCS are non-specific, but we recommend their use in selected cases to exclude generalized neuromuscular conditions and enhance diagnostic accuracy.

Surgical treatment was significantly associated with key outcomes, consistent with existing limited veterinary reports (14, 16, 17). In humans, surgery is the preferred approach, particularly when conservative management fails or clinical deterioration occurs, with reported improvement rates ranging from 70 to 100% (57, 58), aligning with our findings. Surgical detethering has been linked to symptom reversibility, with earlier intervention associated with better outcomes (59). In this cohort, clinical duration did not influence full recovery or treatment requirements; however, surgery increased the likelihood of recovery and reduced ongoing treatment needs. Although some cases with P-OTCS in our cohort responded to medical management, this appeared to be significantly associated with lower clinical severity. While surgery appeared generally effective, three cases experienced recurrence, with two dogs undergoing revision surgery and showing postoperative improvement. Retethering is a recognized complication in humans (60), and these findings underscore the need to optimize current surgical techniques to both address the underlying pathology and minimize the risk of relapse. Vertebral column shortening (VCS) has emerged as an alternative treatment, showing positive results in recurrent or complex TCS cases, with low complication rates (61). However, further studies are needed to compare VCS with traditional detethering.

Surgical success may depend on the portion of the FT transected, though this remains debated. Our study found a potential advantage of transecting both the FT*i* and FT*e* over FT*e* alone, as relapses, incomplete post-operative recovery and ongoing treatment requirements were observed exclusively in dogs undergoing FT*e* dissection alone. While transection of the FT*i* is standard in humans, FT*e* transection alone is less invasive (62), but with controversial efficacy (63). Based on our results, combined transection of FT*i* and FT*e* could be the superior approach for managing OTCS in dogs, addressing the underlying pathology and yielding more favorable long-term outcomes. Nonetheless, statistical comparisons were not performed, and larger studies are needed to investigate these observations.

This study has several limitations that may impact data interpretation and generalizability. Its retrospective, multicentric design introduces variability in clinical practices, subjective descriptions of clinical signs, and non-standardized MRI protocols, complicating consistent identification of anatomical structures. Reliance on retrospective records for intraoperative findings raises concerns about incomplete or misinterpreted data, particularly in the absence of FT histopathology in five surgical cases. For P-OTCS, the lack of surgical confirmation remains a limitation, despite strong clinical suspicion supported by dynamic MRI findings when available and comprehensive exclusion of other conditions. Inconsistent use of dynamic lumbosacral imaging and variation in diagnostic protocols further underscore the presumptive nature of the diagnosis in this group. Clinical signs should be interpreted with caution, as alternative differentials—such as occult spinal dysraphism/myelodysplasia (64), early-stage inherited musculoskeletal diseases (65, 66), peripheral neuropathies (67), and overlooked caudal/sacrocaudal intervertebral disc herniations (68)—must also be considered. The focus on dogs with L4–S3 spinal cord or cauda equina signs may have excluded OTCS cases presenting with upper motor neuron deficits, as reported in human OTCS (21). Broader inclusion criteria in future studies may clarify this aspect. The small sample size limits statistical power and precludes multivariable analyses to adjust for confounders. Additionally, variability and lack of detailed assessment in medical therapy protocols, along with short and inconsistent follow-up durations, further limit the findings. The novel clinical severity score and neurological grading system, while demonstrating significant associations, require external validation in larger, independent cohorts to establish their clinical utility. Despite these limitations, the study provides novel descriptive insights and emphasizes the need for standardized larger prospective studies to enhance diagnostic and therapeutic accuracy.

## 5 Conclusion

This study provides the first comprehensive characterization of canine OTCS, enhancing our understanding of its clinical phenotype, diagnostic features, and treatment strategies. Based on our findings, OTCS appears to be a painful, juvenile-onset condition, with progressive or persistent clinical signs that cannot be explained by any alternative diagnoses and is characterized by a fixed CM on dynamic lumbosacral MRI. Surgical intervention was significantly associated with positive recovery outcomes and reduced the need for prolonged medical management, demonstrating its effectiveness, although relapses may occur. The novel clinical severity scoring and neurological grading systems proved valuable in predicting medical treatment response, offering practical tools for guiding treatment decisions and prognosis assessment. The association between clinical severity score and surgical outcomes warrants investigation. Dynamic lumbosacral MRI showed promise as a diagnostic tool for improving accuracy, while electrodiagnostic techniques require further evaluation. Larger prospective studies are necessary to validate these findings, refine diagnostic criteria, identify robust prognostic factors, and optimize treatment approaches, ultimately improving outcomes for dogs with this challenging condition.

## Data Availability

The original contributions presented in the study are included in the article/Supplementary material, further inquiries can be directed to the corresponding author.
