# Supplementary material for: Occult tethered cord syndrome: insights into clinical and MRI features, prognostic factors, and treatment outcomes in 30 dogs with confirmed or presumptive diagnosis
Source: Front Vet Sci. 2025 Jul 11;12:1588538. doi: 10.3389/fvets.2025.1588538 (PMC12290461; doi:10.3389/fvets.2025.1588538)
Supplement: Supplementary file 7 [file Data_Sheet_1.PDF]

Table S1: Specific behavioral and neurological abnormalities in dogs with OTCS

**Neurological examination***	**T-OTCS** (no. of dogs)	**C-OTCS** (no. of dogs)	**P-OTCS** (no. of dogs)
Behavior	(20)	(10)	(10)
self-mutilation	1	0	1
self-targeted aggression	7	4	3
excessive licking	6	3	3
sudden turning	10	4	6
aversion to touch^	8	6	2
spontaneous vocalization	7	3	4
spontaneous aggression	1	1	0
tail chasing	3	1	2
agitation	14	7	7
subdued demeanor	5	2	3
repetitive sitting	6	3	3
Posture	(10)	(4)	(6)
narrow/wide stance	7	3	4
lumbar kyphosis	2	1	1
pelvic limb weight shifting	3	0	3
low-tail carriage	4	3	1
Gait	(21)	(9)	(12)
paresis	5	3	2
ataxia	0	0	0
abduction	3	1	2
stiffness	10	5	5
lameness	11	4	7
bunny-hopping	3	1	2
difficulty jumping/climbing stairs	14	4	10
Involuntary movements	(3)	(1)	(2)
tremor	3	1	2
Postural reactions	(7)	(4)	(3)
hopping	5	3	2
paw repositioning	4	2	2
Spinal segmental reflexes	(11)	(6)	(5)
withdrawal	11	6	5
patellar	1	0	1
perineal	3	1	2
Muscle mass/tone	(10)	(3)	(7)
reduced mass	10	3	7
reduced tone	3	2	1
Spinal palpation	(24)	(10)	(14)
lumbosacral	24	10	14
tail	7	4	3

T-OTCS = entire cohort of dogs with OTCS; C-OTCS = surgically managed confirmed OTCS group; P-OTCS = medically managed presumptive OTCS group.
*focused on pelvic limbs and lumbosacral/tail region
^including behaviors such as aggression and vocalization to touch
