# Supplementary material for: Occult tethered cord syndrome: insights into clinical and MRI features, prognostic factors, and treatment outcomes in 30 dogs with confirmed or presumptive diagnosis
Source: Front Vet Sci. 2025 Jul 11;12:1588538. doi: 10.3389/fvets.2025.1588538 (PMC12290461; doi:10.3389/fvets.2025.1588538)
Supplement: Supplementary file 8 [file Data_Sheet_2.PDF]

Table S2: Prevalence of clinical signs used in clinical severity scoring system in dogs with OTCS

Clinical signs	T-OTCS (no. of dogs)	C-OTCS (no. of dogs)	P-OTCS (no. of dogs)
**Neurological deficits***	27	11	16
Posture	10	4	6
Gait	21	9	12
Postural reactions	7	4	3
Spinal segmental reflexes	11	6	5
Muscle mass/tone	10	3	7
**Pain^**	29	11	18
Lumbosacral/tail base	26	10	16
Pelvic limb/s	8	4	4
During defecation/urination	7	4	3
**Incontinence**	5	4	1
Fecal	2	1	1
Urinary	4	4	0
**Behavior**	20	10	10
Aggression	7	5	2
Compulsion	9	5	4
Restlessness	8	5	3
Lethargy	7	4	3
**Physical activity**	19	8	11
Intolerance	7	3	4
Reluctance**	12	5	7

T-OTCS = entire cohort of dogs with OTCS; C-OTCS = surgically managed confirmed OTCS group; P-OTCS = medically managed presumptive OTCS group.

*in pelvic limbs

^including episodic or continuous suspected dysesthesia

**able to exercise but unwilling / reluctant to do so
