# Supplementary figures and images for: Occult tethered cord syndrome: insights into clinical and MRI features, prognostic factors, and treatment outcomes in 30 dogs with confirmed or presumptive diagnosis
Source: Front Vet Sci. 2025 Jul 11;12:1588538. doi: 10.3389/fvets.2025.1588538 (PMC12290461; doi:10.3389/fvets.2025.1588538)

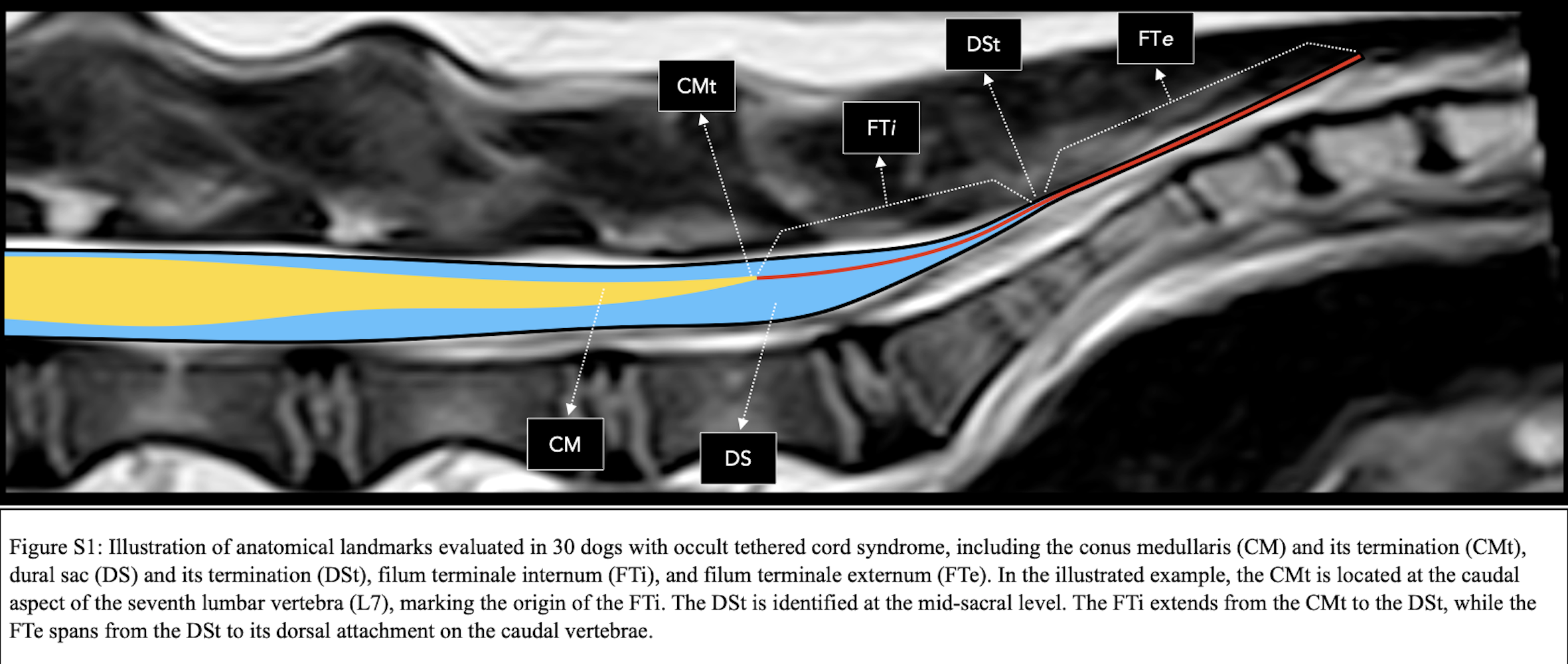

Supplement: Supplementary file 9 [file Image_1.TIFF]
